# Shiga Toxin-Bearing Microvesicles Exert a Cytotoxic Effect on Recipient Cells Only When the Cells Express the Toxin Receptor

**DOI:** 10.3389/fcimb.2020.00212

**Published:** 2020-05-25

**Authors:** Karl Johansson, Annie Willysson, Ann-Charlotte Kristoffersson, Ashmita Tontanahal, Daniel Gillet, Anne-lie Ståhl, Diana Karpman

**Affiliations:** ^1^Department of Pediatrics, Clinical Sciences Lund, Lund University, Lund, Sweden; ^2^Université Paris-Saclay, CEA, INRAE, Médicaments et Technologies pour la Santé, (MTS), SIMoS, Gif-sur-Yvette, France

**Keywords:** Shiga toxin, enterohemorrhagic *Escherichia coli*, hemolytic uremic syndrome, microvesicles, globotriaosylceramide, Gb3, retrograde transport

## Abstract

Shiga toxin is the main virulence factor of non-invasive enterohemorrhagic *Escherichia coli* strains capable of causing hemolytic uremic syndrome. Our group has previously shown that the toxin can reach the kidney within microvesicles where it is taken up by renal cells and the vesicles release their cargo intracellularly, leading to toxin-mediated inhibition of protein synthesis and cell death. The aim of this study was to examine if recipient cells must express the globotriaosylceramide (Gb3) toxin receptor for this to occur, or if Gb3-negative cells are also susceptible after uptake of Gb3-positive and toxin-positive microvesicles. To this end we generated Gb3-positive A4GALT–transfected CHO cells, and a vector control lacking Gb3 (CHO-control cells), and decreased Gb3 synthesis in native HeLa cells by exposing them to the glycosylceramide synthase inhibitor PPMP. We used these cells, and human intestinal DLD-1 cells lacking Gb3, and exposed them to Shiga toxin 2-bearing Gb3-positive microvesicles derived from human blood cells. Results showed that only recipient cells that possessed endogenous Gb3 (CHO-Gb3 transfected and native HeLa cells) exhibited cellular injury, reduced cell metabolism and protein synthesis, after uptake of toxin-positive microvesicles. In Gb3-positive cells the toxin introduced via vesicles followed the retrograde pathway and was inhibited by the retrograde transport blocker Retro-2.1. CHO-control cells, HeLa cells treated with PPMP and DLD-1 cells remained unaffected by toxin-positive microvesicles. We conclude that Shiga toxin-containing microvesicles can be taken up by Gb3-negative cells but the recipient cell must express endogenous Gb3 for the cell to be susceptible to the toxin.

## Introduction

Shiga toxin 2 (Stx2) is an AB5 toxin that consists of a pentameric B-subunit, which mediates binding, and an enzymatically active A-subunit (Endo et al., 1988; Ling et al., 1998). Stx2 binds to the glycosphingolipid globotriaosylceramide (Gb3) or globotetraosylceramide (Gb4) in order to gain entry to cells (Jacewicz et al., 1986; Gallegos et al., 2012). Once inside the cell, Stx2 is transported via the retrograde pathway to the endoplasmic reticulum (ER) (Sandvig et al., 1992). Along the transport route the A-subunit is enzymatically cleaved into A_1_- and A_2_-moieties that are held together via disulphide bonds (Garred et al., 1995). In the ER the disulphide bonds are reduced and the A_1_-moiety is released into the cytosol, where it depurinates a specific base in the 28S rRNA of the ribosome, inhibiting protein translation, ultimately leading to cell death (Sandvig and van Deurs, 1996; Spooner and Lord, 2012).

Stx2 is the main virulence factor of enterohemorrhagic *Escherichia coli* (EHEC). EHEC is a food-borne human pathogen that colonizes the large intestine, causing diarrhea and hemorrhagic colitis, and in severe cases hemolytic uremic syndrome (HUS) that may lead to acute kidney injury and death (Tarr et al., 2005). EHEC is a non-invasive bacterium that secretes virulence factors, including Stx2, that gain access to the circulation (McKee and O'Brien, 1995). Stx2 binds to blood cells and is taken up (Falguieres et al., 2001; Karpman et al., 2001). The blood cells shed Stx2-containing microvesicles (Ståhl et al., 2009, 2015). We have previously shown that these Stx2-positive blood cell-derived microvesicles circulate in EHEC-infected patients and in EHEC-infected mice (Ståhl et al., 2015). The toxin is thereby transported in the systemic circulation and the microvesicles, with their toxic cargo, are taken up by kidney cells (Karpman et al., 2017). Once intracellular the toxin is released from the microvesicles and leads to inhibited protein synthesis (Ståhl et al., 2015).

Stx2-positive microvesicles were taken up in murine glomerular endothelium in the EHEC infection model (Ståhl et al., 2015). Mouse glomerular endothelial cells are Gb3-negative (Psotka et al., 2009), providing evidence for microvesicle-mediated Stx2-uptake in cells lacking endogenous Gb3. This prompted the current study in which we aimed to investigate if the presence of Gb3 in microvesicles is sufficient for the induction of toxin-mediated cellular injury or if the recipient cell must also possess the Gb3 receptor for this to occur.

To this end we investigated the effect of Stx2 delivered within microvesicles on Gb3-positive and Gb3-negative cells. We used Chinese hamster ovary (CHO) cells that are inherently Gb3-negative and generated Gb3-positive transfected CHO cells. We decreased Gb3 synthesis in HeLa cells using a glycosylceramide synthase inhibitor and also used DLD-1 human intestinal cells, naturally lacking Gb3. Cells were incubated with Gb3-positive Stx2-positive microvesicles. The intracellular transport route of Stx2 delivered via microvesicles was investigated. The specific goal was to determine if the presence of Gb3 in recipient cells was essential for cytotoxicity of Stx2 delivered within microvesicles.

## Methods

### Shiga Toxin

Stx2a was purchased from Phoenix Lab (Tufts Medical Center, Boston, MA). Lipoplysaccharide (LPS) contamination was measured using the Limulus Amebocyte Lysate method (Thermo Fisher Scientific, Rockford, IL) detecting minute amounts (183.4 ng/mg toxin). For certain experiments Stx2 was labeled with Alexa Fluor 488 or Alexa Fluor 555 using the Microscale Protein Labeling Kit (both from Thermo Fisher Scientific) according to the manufacturer's instructions. The toxic activity of Stx2 was retained after labeling with fluorescent dyes, as determined by the cell metabolism assay described below.

### Generation of Blood Cell-Derived Stx2-Containing Microvesicles

Human whole blood was drawn from healthy volunteers (*n* = 5, 24 mL from each) into citrated blood collection tubes (Becton Dickinson, Franklin lanes, NJ), diluted 1:1 with DMEM (Gibco, Waltham, MA) containing glycin-proline-arginine-proline peptides (GPRP, 1 mM, Sigma-Aldrich, Steinheim, Germany), to prevent fibrin polymerization, and incubated with Stx2 (final concentration of 200 ng/mL) or phosphate buffered saline (PBS, GE Life Sciences, Chicago, IL) for 40 min at 37°C under gentle rocking. The blood was centrifuged at 1,500 × g for 15 min and the supernatant, containing platelet-poor plasma, was collected and centrifuged at 10,000 × g for 10 min. The supernatant, containing microvesicles, was collected, washed thrice with PBS and centrifuged at 20,000 × g for 40 min. Microvesicles were pooled, aliquoted, and stored at −80°C until used. The microvesicles were analyzed for Stx2-content and size distribution as described below. DMEM and PBS were filtered through 0.2 μm pore-sized filters (Pall Corporation, Ann Arbor, MI) to remove aggregates. The microvesicles were used in the cell metabolism and protein synthesis assays described below.

### Determination of Stx2 Concentration in Blood Cell-Derived Microvesicles

The Stx2 content of the microvesicle suspension and the supernatant that was removed from the last wash from the microvesicle supernatant was determined by ELISA. White 96-well Maxisorp plates (Nunc, Roskilde, Denmark) were coated with mouse anti-Stx2 (1 μg/mL, 11E10, Hycult Biotech, Uden, Netherlands) suspended in 0.1 M carbonate buffer with pH 9.6 (Merck, Darmstadt) overnight at 4°C. Wells were washed thrice with PBS Tween 0.05% (PBS-T, Medicago, Uppsala, Sweden) and blocked with bovine serum albumin (1%, BSA, Sigma-Aldrich) in PBS for 1 h at rt. Microvesicle suspensions and supernatants were incubated with saponin. Saponin (0.5%, Sigma-Aldrich) was used to permeabilize the microvesicles to enable antibody detection of intravesicular Stx2. Microvesicle samples and a standard curve of Stx2 (range 500–1.6 ng/mL), all diluted in BSA, were incubated overnight at rt. Wells were washed with PBS-T thrice and incubated with rabbit anti-Stx2 (1:1,000, BEI Resources, Manassas, VA) diluted in BSA for 1 h at rt. Wells were washed with PBS-T as above and incubated with goat anti-rabbit HRP (1:1,000, Dako, Glostrup, Denmark), in BSA. Wells were washed and developed using SuperSignal ELISA Pico (Thermo Fisher Scientific) according to the manufacturer's instructions. Luminescence was measured using a Glomax Discover System (Promega, Madison, WI) at 1 s integration time per well. The Stx2 concentration of the saponin-treated microvesicle suspension was 30.3 ng/mL. No toxin was detected in the supernatant from the microvesicle suspension. The percentage of the recovered toxin in the microvesicle suspension was 0.13% of the initial toxin dose after adjusting for volumes.

### Size Distribution of Blood Cell- Derived Microvesicles

Microvesicle samples were analyzed for size distribution using nanoparticle tracking analysis. Briefly, samples were diluted 1:10 in PBS filtered through 0.2 μm pore-size filters and loaded into a syringe pump. The microvesicle suspensions were recorded under flow using a NanoSight LM10 instrument equipped with a 405 nm laser (NanoSight, Amesbury, UK). Three consecutive recordings were analyzed with NTA software 3.2 (NanoSight) giving a peak at 124.3 nm (range 56.5–672.5 nm) for Stx2-positive microvesicles and 133.7 nm (range 41.5–808.5) for Stx2-negative microvesicles. These data suggest that most of the vesicles were shed microvesicles (100–1,000 diameter) but the presence of some exosomes (30–100 nm) could not be ruled out.

### Cell Cultures

Chinese hamster ovary (CHO-native) epithelial cells (ATCC, Manassas, VA), DLD-1 (colonic epithelial) cells (ATCC), or HeLa cells (cervical epithelial, a kind gift from L. Johannes, Institute Curie, Paris) were cultured in DMEM supplemented with 10% fetal calf serum and 1% penicillin-streptomycin (both from Gibco) in 5% CO_2_ at 37°C.

### Stable Gb3-Expressing CHO Cells

CHO-native cells were transfected with the pEF1α-IRES-ZsGreen1 plasmid (CloneTech Laboratories, Mountain View, CA) containing A4GALT cDNA or the corresponding control vector (a kind gift from Martin L. Olsson, Transfusion Medicine, Lund University), lacking the cDNA insert, using Lipofectamine 3000 (Thermo Fisher Scientific). Two days after transfection G418 (400 μg/mL, Sigma-Aldrich) was added to the cells to select for transfected clones. After 7 days of selection cells were seeded out at a density of 1 cell per well in a 96-well plate (Corning, Corning, NY). Clones were picked based on the presence of ZsGreen and Stx2:Alexa555 binding in A4GALT-positive CHO cells (CHO-Gb3) or a lack of Stx2:Alexa555 binding in the vector control CHO cells (CHO-control).

### Gb3-Silencing in HeLa Cells Using a CD77 Synthase shRNA Plasmid

HeLa cells were transfected with the CD77 synthase shRNA plasmid or with the control shRNA Plasmid A using Plasmid Transfection Reagent (all from Santa Cruz Biotechnology, Dallas, TX). Two days after transfection puromycin (2 μg/mL, Sigma-Aldrich) was added to the cells to select for transfected clones. Two clones from the cells transfected with the CD77 synthase shRNA plasmid and two clones from the cells transfected with the control plasmid were isolated and further cultured. The clones were analyzed for the presence of Gb3 by glycosphingolipid extraction and separation using thin layer chromatography followed by visualization by orcinol staining, as described below. Results showed that reduction in Gb3 had not been obtained and therefore inhibition of Gb3 synthesis was pursued as described below.

### Inhibition of Gb3 Synthesis in HeLa Cells

HeLa cells were treated with the glycosylceramide synthase inhibitor D-threo-1-Phenyl-2-hexadecanoylamino-3-morpho lino-1-propanol (PPMP 5 μM, Abcam, Cambridge, UK) for at least 10 days to inhibit synthesis of Gb3.

### Lipid Extraction, Thin Layer Chromatography and Stx2-Overlay

CHO-native, CHO-Gb3 (expressing Gb3), CHO-control (the vector control), HeLa cells, PPMP-treated HeLa cells and DLD-1 cells were grown in two T75 cell culture flasks (Thermo Fisher Scientific) each. Once confluent cells were treated with trypsin and washed twice in PBS, by centrifugation at 500 × g for 10 min.

Microvesicles were purified, as described above, from 12 mL human whole blood drawn from healthy volunteers into citrated blood collection tubes (Becton Dickinson), diluted 1:1 with DMEM and incubated with calcium ionophore A23187 (10 μM, Sigma-Aldrich), to stimulate microvesicle release, for 40 min at 37°C.

Lipid extraction and thin layer chromatography (TLC) of cells and microvesicles was performed as described previously (Hedlund et al., 1996) with some modifications. Lipids were visualized by orcinol staining (Supplementary Figures 1A–D) and for microvesicles also by Stx2-binding overlay (Supplementary Figure 2). For Stx2-binding overlay, the TLC plate with separated lipids was immersed in P28 (Poly(ethyl methacrylate), blocked in 1% BSA for 1 h and incubated with Stx2 200 ng/mL for 1 h. The TLC plate was washed with TRIS-buffered saline ×3 and incubated with mouse anti-Stx2 (1 μg/mL) for 1 h. The TLC plate was washed again and incubated with anti-mouse HRP (1:1,000) followed by visualization with Pierce ECL immunoblotting substrate (Thermo Fisher Scientific) (Supplementary Figure 2). All chemicals were purchased from Sigma-Aldrich, unless otherwise indicated.

### Cell Metabolism Assay

Cells (15,000 CHO-Gb3 or CHO-control or 10,000 DLD-1 cells/well) were seeded out in black 96-well plates (Corning) with clear bottoms 24 h before the start of the experiments. Cells were incubated with blood cell-derived Stx2-positive microvesicles (final Stx2 concentration/well: 2 ng/mL as determined by ELISA), Stx2-negative microvesicles (CHO-Gb3 or CHO-control), free Stx2 (2 ng/mL CHO-Gb3, CHO-control or DLD-1 cells), or PBS alone (CHO-Gb3, CHO-control or DLD-1), all diluted in serum-free DMEM (Gibco). The amount of microvesicles in Stx2-positive microvesicles and Stx2-negative microvesicles was equalized based on protein content, as measured by light absorption at 280 nm (NanoDrop, Thermo Fisher Scientific). After 24 h incubation cells were washed twice with PBS and Alamar Blue (Thermo Fisher Scientific) diluted 1:10 in serum-free DMEM was added to the cells. The time-point of 24 h was chosen as all cells detached after longer incubations. When a clear shift in the dye was visible the plates were read in a Glomax Discover System (Promega), using 520 nm excitation light and 580–640 nm emission filters. Using this assay we could demonstrate that the CHO-Gb3 cells were sensitive to free Stx2 whereas the CHO-control (vector control) cells were not (Supplementary Figure 3).

Using the same method a dose response assay was designed to determine the effect of Stx2-positive microvesicles vs. free Stx2 on cell metabolism. For these experiments Stx2-positive microvesicles were added at concentrations between 0.015 and 3.75 ng/mL and free Stx2 between 0.007 and 3.75 ng/mL. The IC_50_ value of Stx2-positive microvesicles was 0.088 ng/mL and the IC_50_ value of free Stx2 was 0.11 ng/mL (Supplementary Figure 4). The cell metabolism assay was not performed using HeLa and PPMP-treated HeLa cells.

### Protein Synthesis Assay

CHO-Gb3 or CHO-control cells (15,000 cells/well) or HeLa, HeLa-PPMP, or DLD-1 cells (10,000 cells/well) were seeded out and cultured in black 96-well plates with clear bottoms (Corning) 24 h before incubation with blood cell-derived Stx2-positive microvesicles, Stx2-negative microvesicles, free Stx2 (2 or 3 ng/mL), or PBS alone, as described above. In certain experiments CHO-Gb3 cells were pre-treated with Retro-2.1. Retro-2.1 is an early-endosome-to-Golgi-transport inhibitor that has been shown to protect cells from Stx2-induced protein synthesis inhibition (Gupta et al., 2014). Retro-2.1 (1 μM) was added for 30 min before addition of blood cell-derived Stx2-positive microvesicles or free Stx2 and was maintained throughout the experiment. Cells were incubated with microvesicles, free Stx2 or PBS for a total of 3 h and 15 min, washed with PBS three times and incubated with L-homopropargylglycine (HPG, 1:1,000) diluted in methionine-free RPMI medium (Gibco) for 45 min before protein synthesis was measured using the Click-iT HPG Protein Synthesis Assay Kit (Thermo Fisher Scientific) according to the manufacturer's instructions. In total, cells were exposed to Stx2 for 4 h. This time point was selected as a previous study showed that Stx localized to the ER after 2 h (Johansson et al., 2019). Protein synthesis measurements were carried out using Alexa Fluor 647 azide for CHO-Gb3 and CHO-control cells or Alexa Fluor 488 azide for HeLa cells and DLD-1 cells. Cell fluorescence was measured using the Glomax Discover System and DAPI filter sets (excitation 365 nm, emission 415–445 nm) for NuclearMask Blue stain and far-red filter settings (excitation 627 nm, emission 660–720 nm) for CHO-Gb3 and CHO control cells or FITC filter settings (excitation 475 nm, emission 500–550 nm) for HeLa cells and DLD-1 cells. Results per cell are presented as protein synthesis divided by the DAPI nuclear stain. Retro-2.1 had a protective effect on CHO-Gb3 cells exposed to free Stx2 as indicated by protein synthesis: 15% of untreated cells in the presence of free Stx2 compared to 33.2% when co-incubated with Retro-2.1.

### Quantification of Microvesicle Uptake

CHO-Gb3 or CHO-control cells (15,000 cells/well) were seeded out and cultured in 96-well plates 24 h before incubation with blood cell-derived microvesicles. After 2 or 24 h the microvesicle-suspensions that had been incubated with the cells and microvesicle suspensions that had not been in contact with cells, were collected and analyzed by a CD41/CD61 ELISA (Novus Biologicals, Centennial, CO), according to the manufacturer's instructions. Absorbance was measured at 450 nm using the Glomax Discover System.

### Generation of Fluorescent Platelet-Derived Stx2:Alexa555-Containing Microvesicles

Whole blood was drawn as described above and centrifuged at 200 × g for 15 min. The platelet-rich supernatant was collected and further centrifuged at 2,000 × g for 10 min to obtain a pellet. The supernatant was discarded, and platelets were suspended and stained in 5 μM CellTrace CFSE (Thermo Fisher Scientific) diluted in washing buffer (NaCl 140 mM, EDTA 9 mM, Na_2_HPO_4_ 26 mM, adjusted to pH 7) for 30 min in the dark. Platelets were washed thrice in washing buffer and resuspended in cold Hank's Balanced Salt Solution with Ca^2+^/Mg^2+^ (HBSS), containing Stx2 labeled with Alexa 555 (200 ng/mL, final concentration) and GPRP (1 mM) for 1 h in 4°C and washed twice in cold washing buffer. Platelets were resuspended in cold HBSS and stimulated with calcium ionophore A23187 (10 μM final concentration) for 40 min at 37°C followed by centrifugation at 10,000 × g for 10 min. The microvesicle-containing supernatant was collected and further washed as described above. These microvesicles were used for detection of microvesicle uptake by CHO-native cells described below.

### Generation of Fluorescent HeLa Cell-Derived Stx2:Alexa488- Containing Microvesicles

HeLa cells were seeded out at a density 150,000 cells/mL in Fluorobrite DMEM media (Gibco) in T75 culture flasks (Thermo Fisher Scientific). One day later the cells were incubated with TNF-α (20 ng/mL, Sigma-Aldrich) at 37°C for 24 h. Cells were washed twice in PBS and incubated in ice-cold FluoroBrite DMEM media with Stx2:Alexa 488 (200 ng/mL, final concentration) for 1 h in 4°C. Cells were washed as above and incubated with A23187 10 μM for 40 min to induce microvesicle release. The cell media was collected and centrifuged at 2,500 × g for 5 min and the supernatant, containing microvesicles, was collected and washed as described above. These microvesicles were used for detection and quantification of microvesicle uptake by DLD-1 cells.

### Microvesicle Uptake Assay

Cells (CHO-native (45,000) or DLD-1 (30,000) cells/well) were seeded out in an Ibidi 8-well chamber slide (Ibidi, Gräfelfing, Germany) 1 day before the start of the experiment. CHO-native cells were washed twice with PBS and incubated with the platelet-derived microvesicle suspension derived from a total of 1.2 ml of whole blood or Stx2:Alexa555 200 ng/mL, corresponding to the concentration used to generate the Stx2-positive platelet microvesicles.

DLD-1 cells were incubated with the microvesicle suspension derived from 350,000 HeLa cells, or Stx2:Alexa 488 at 31 ng/mL, which gave the equivalent fluorescence as the microvesicle suspension when measured in the Glomax Discover System.

Microvesicles and labeled Stx2 were diluted in Opti-MEM (Invitrogen, Carlsbad, CA) and incubated with the cells for 4 h. After the incubation, cells were fixed in 4% paraformaldehyde (Histolabs, Västra Frölunda, Sweden), for 20 min. CHO-native cells were stained with NucBlue Live Cell Stain (Thermo Fisher Scientific) and DLD-1 cells were stained with Cellmask Deep Red plasma membrane stain (1.6 μg/mL, Thermo Fisher Scientific) and visualized in an Axio Observer.A1 inverted fluorescence microscope (Zeiss) or in a Ti-E inverted fluorescence microscope equipped with a Nikon structured illumination microscopy module (Nikon Instruments, Tokyo, Japan).

### Quantification of Stx2:Alexa488 in DLD-1 Cells

Images captured using fluorescence microscopy of DLD-1 cells that had been incubated with HeLa cell-derived Stx2:Alexa488-positive microvesicles or free toxin were quantified using ImageJ (ImageJ version 1.52n, Bethesda, MA). Each image stack was converted to a max intensity image. Individual cells were outlined and a minimum threshold for green fluorescence was set to 2100 pixel intensity. The area of the green pixels in each cell was quantified and plotted (Supplementary Figure 5).

### Exogenous Gb3 Administration

Gb3 (200 μg, Matreya LLC, State College, PA), phosphatidylethanolamine (Larodan, Solna, Sweden), and phosphatidylserine (Sigma-Aldrich) diluted in methanol:chloroform (16:13) were mixed and dried under a stream of nitrogen. The lipids were resuspended in PBS 400 μL, vortexed (4 × 30 s, setting 10, Vortex Genie 2, Scientific Industries, Bohemia, NY) and sonicated in a water-bath (Metler Electronics, Anaheim, CA) for 30 min to form liposomes. The Gb3-containing liposomes were immediately added to CHO-native or DLD-1 cells at a concentration of 3.75 μg/100,000 cells. To verify that the liposomes were able to bind Stx2, some of the liposome suspension was added to a chamber slide (Ibidi) in the absence of cells together with Stx2:Alexa 488 200 ng/mL or PBS and imaged in an Axio Observer A1 inverted fluorescence microscope (Supplementary Figure 6). Liposomes were incubated with cells for 1 h, washed twice in PBS and incubated in complete DMEM for 24 h before they were used in cell metabolism assays or for microscopy. For microscopy experiments CHO-native or DLD-1 cells treated with Gb3-liposomes were incubated with Stx2:Alexa 488 for 1 h in a cell incubator followed by 3 × wash with PBS. The cells were imaged using an Axio Observer A1 inverted fluorescence microscope (Zeiss, Oberkochen, Germany) (Supplementary Figure 7 showing DLD-1 cells, similar results were obtained using CHO-native cells, data not shown).

### Statistical Analysis

For comparison between two groups the two-tailed Mann-Whitney *U*-test was used and for more than two groups the Kruskal-Wallis multiple-comparison test was used followed by comparison between specific groups using the Dunn procedure. Statistical analyses were performed using Prism 7 version 7.0a (GraphPad, La Jolla, CA).

## Results

### Stx2-Positive Microvesicles Induce Toxicity in Gb3-Positive but Not in Gb3- Negative Cells

**Figure 1:**
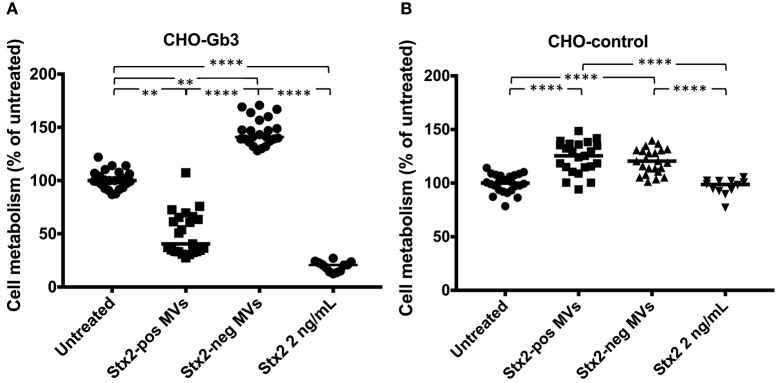
Gb3-positive but not Gb3-negative CHO cells are susceptible to microvesicle-delivered Stx2. CHO-Gb3 and CHO-control cells were incubated with Shiga toxin 2-positive microvesicles (Stx2-pos MVs), Stx2-negative microvesicles (Stx2-neg MVs), free Stx2 or PBS and cell metabolism was measured. **(A)** CHO-Gb3 cells were treated with Stx2-pos microvesicles (*n* = 23), Stx2-negative microvesicles (*n* = 23), free Stx2 (*n* = 12), or PBS (*n* = 24). Stx2-positive microvesicle-treated and free Stx2-treated cells exhibited a significantly lower cell metabolism compared to cells treated with Stx2-negative microvesicles and untreated cells. **(B)** CHO-control cells were treated with Stx2-positive microvesicles (*n* = 23), Stx2-negative microvesicles (*n* = 23), free Stx2 (*n* = 12), or PBS (*n* = 24). Cells treated with Stx2-positive microvesicles or Stx2-neg microvesicles had a significantly higher cell metabolism compared to untreated and Stx2-treated cells. No difference between Stx2-treated and untreated CHO-control cells was found. Median cell metabolism is denoted by the bar. **P < 0.01, ****P < 0.0001, Kruskal-Wallis test. Two independent experiments are presented.

**Figure 2:**
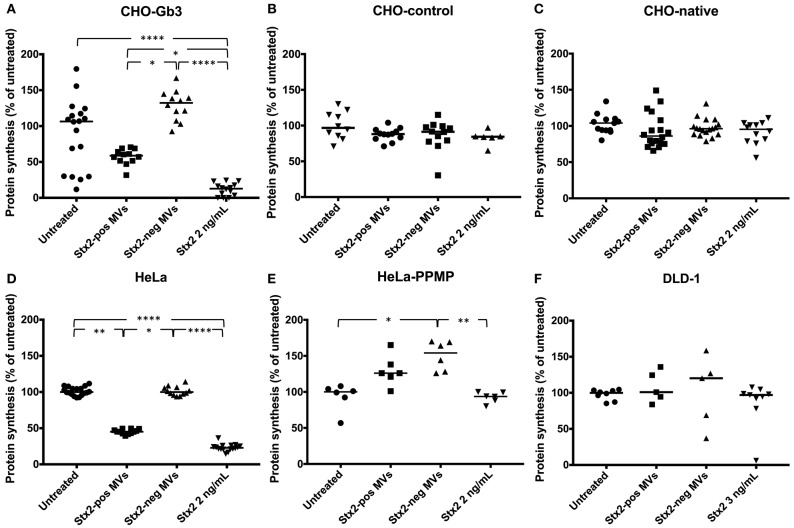
Stx2-positive microvesicles are toxic to Gb3-positive but not Gb3-negative cells. CHO-Gb3, CHO-control and CHO native cells, HeLa and HeLa-PPMP and DLD-1 cells were incubated with Shiga toxin 2 positive microvesicles (Stx2-pos MVs), Stx2-negative microvesicles (Stx2-neg MVs), Stx2 or PBS and protein synthesis was measured. **(A)** CHO-Gb3 cells were incubated with Stx2-positive microvesicles (*n* = 12), Stx2-negative microvesicles (*n* = 12), free Stx2 (*n* = 14), or PBS (*n* = 18). A significant decrease in protein synthesis was seen in both Stx2-positive microvesicles and free Stx2 compared to Stx2-negative microvesicles (2 independent experiments). **(B)** CHO-control cells were treated with Stx2-positive microvesicles (*n* = 12), Stx2-negative microvesicles (*n* = 12), free Stx2 (*n* = 6), or PBS (*n* = 10). No differences in protein synthesis were noted between the groups (one experiment). **(C)** CHO native cells were incubated with Stx2-positive microvesicles (*n* = 18), Stx2-negative microvesicles (*n* = 18), free Stx2 (*n* = 12), or PBS (*n* = 13). No differences in protein synthesis were noted between the groups (three independent experiments). **(D)** HeLa cells were incubated with Stx2-positive microvesicles (*n* = 12), Stx2-negative microvesicles (*n* = 12), free Stx2 (*n* = 16), or PBS (*n* = 18). Cells treated with Stx2-positive microvesicles and free Stx2 exhibited significantly lower protein synthesis compared to untreated and Stx2-negative microvesicle-treated cells (two independent experiments). **(E)** HeLa-PPMP cells were treated with Stx2-positive microvesicles (*n* = 6), Stx2-negative microvesicles (*n* = 6), free Stx2 (*n* = 6), or PBS (*n* = 6). No difference in protein synthesis was seen between the groups (one experiment). **(F)** DLD-1 cells were incubated with Stx2-positive microvesicles (*n* = 5), Stx2-negative microvesicles (*n* = 5), free Stx2 (*n* = 9), or PBS (*n* = 8). No differences in protein synthesis were seen between the groups (two independent experiments). Median protein synthesis per cell is denoted by the bar. The median protein synthesis of untreated cells was defined as 100% in each experiment. *P < 0.05, **P < 0.01, ****P < 0.0001, Kruskal-Wallis test.

We transfected CHO-native cells that thereby express Gb3 and inhibited synthesis of Gb3 from HeLa cells using PPMP. CHO-Gb3 and CHO-control cells were incubated with blood cell-derived Stx2-positive and Stx2-negative microvesicles or free toxin, at the same concentration as in Stx2-positive microvesicles, for 24 h. CHO-Gb3 cells incubated with Stx2-positive microvesicles exhibited a significantly lower cell metabolism compared to cells incubated with Stx2-negative microvesicles (Figure 1A, Table 1), while in CHO-control cells no effect or significant difference between the same groups could be demonstrated (Figure 1B). The cell metabolism of CHO-Gb3 cells treated with Stx2-positive microvesicles was significantly lower compared to CHO-control cells treated with these microvesicles (*P* < 0.0001). The effect of the blood cell-derived Stx2-positive and Stx2-negative microvesicles or free toxin on protein synthesis was examined using CHO-Gb3, CHO-control, CHO native, HeLa wild-type cells, HeLa-PPMP treated cells, and DLD-1 cells. Results showed that CHO-Gb3 cells incubated with Stx2-positive microvesicles for 4 h had a significant decrease in protein synthesis compared to those incubated with Stx2-negative microvesicles (Figure 2A), in contrast to CHO-control and CHO native cells that displayed no difference in protein synthesis (Figures 2B,C). Similarly, HeLa cells incubated with Stx2-positive microvesicles for 4 h resulted in a significant reduction in protein synthesis while PPMP-treated HeLa cells were unaffected (Figures 2D,E). DLD-1 cells that were incubated with Stx2-positive microvesicles did not exhibit any changes in protein synthesis (Figure 2F). CHO-Gb3 and HeLa cells that had been treated with Stx2-positive microvesicles had a significantly lower protein synthesis compared to CHO-control cells and PPMP-treated HeLa cells (*P* < 0.0001 and *P* < 0.001, respectively). These results indicate that the presence of Gb3 in the recipient cell affects protein synthesis inhibition induced by Gb3-positive Stx2-bearing microvesicles.

**Table 1:** Cell metabolism and protein synthesis in cells exposed to Stx2-positive microvesicles and free toxin.

**Experiments**		**CHO cells**			**HeLa cells^a^**		**DLD-1 cells^b^**
		**CHO-control^b^ (vector)**	**Gb3-positive**			**PPMP^b^**	
				**Retro-2.1**			
Stx2-pos MVs	Cell metabolism	UC^c^	↓↓^c^	NA	NA	NA	NA
	Protein synthesis	UC^d^	↓↓^d^^,^^e^	↓^e^	↓↓^d^	UC^d^	UC^d^
Free Stx2	Cell metabolism	UC^c^	↓↓^c^	NA	NA	NA	UC^f^
	Protein synthesis	UC^d^	↓↓^d^	NA	↓↓^d^	UC^d^	UC^d^

a *Gb3-positive cells*;

b *Gb3-negative cells*;

c *Values are presented in Figure 1*;

d *Values are presented in Figure 2*;

e *Values are presented in Figure 3*;

f *Values are presented in Figure 5; ↓, Lowered cell metabolism/viability or protein synthesis; CHO, Chinese hamster ovary; DLD-1, intestinal epithelial cell line; Gb3, Globotriaosylceramide; PPMP, D-threo-1-Phenyl-2-hexadecanoylamino-3-morpholino-1-propanol; Stx2-pos MVs, blood cell-derived Stx2-positive microvesicles; UC, Unchanged; NA, Not assayed*.

### Microvesicle-Delivered Stx2 Is Transported via the Retrograde Pathway

To investigate the intracellular transport route of Stx2 delivered via microvesicles, CHO-Gb3 cells were treated with the retrograde transport inhibitor Retro-2.1 or PBS vehicle for 30 min before addition of Stx2-positive microvesicles. Results showed that the protein synthesis associated with microvesicle-delivered Stx2 was lower in the cells that had been treated with PBS compared to the Retro-2.1-treated cells (Figure 3). These results indicate that Stx2 within microvesicles affects protein synthesis by the same retrograde route as free toxin and is in line with a previous publication showing that Stx2 delivered via microvesicles reaches the ER (Ståhl et al., 2015).

**Figure 3:**
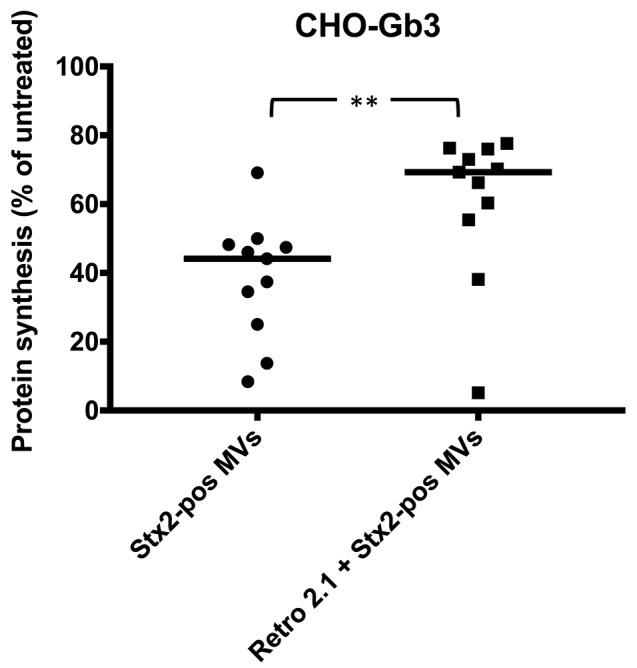
Shiga toxin 2 delivered by microvesicles utilizes the intracellular retrograde pathway. The intracellular pathway of Shiga toxin 2-positive microvesicles (Stx2-pos MVs) was determined using Retro-2.1. CHO-Gb3 cells were treated with Retro-2.1 or PBS before addition of Stx2-positive microvesicles (Stx2-pos MVs) (*n* = 11). A significantly higher protein synthesis was observed in CHO-Gb3 cells treated with Retro-2.1 compared to cells without pretreatment (median 69.3 and 44.1%, respectively, compared to untreated cells, defined as 100%). Median protein synthesis per cell is denoted by the bar. **P < 0.01, Two-tailed Mann-Whitney *U*-test. Two independent experiments are presented.

### Gb3-Negative CHO-native and DLD-1 Cells Can Take Up Stx2 Delivered by Microvesicles as Determined by Microscopy

**Figure 4:**
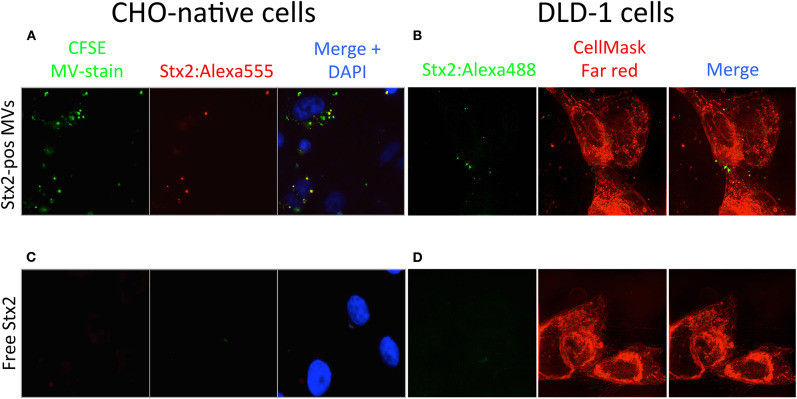
Shiga toxin 2 can be taken up by Gb3-negative cells within microvesicles. **(A)** CHO-native cells were incubated with CFSE-stained microvesicles (green dye) containing Stx2:Alexa555 (Stx2-pos MVs, red dye) and stained with DAPI. Fluorescence microscopy showed that microvesicles were associated with CHO-native cells and that some of the microvesicles were positive for Stx2:Alexa555. **(B)** DLD-1 cells were incubated with microvesicles positive for Stx2:Alexa488 (Stx2-pos MVs, green dye). 3D-SIM imaging showed that fluorescence corresponding with Stx2:Alexa488 was present within DLD-1 cells (as determined by z-stack acquisition) when delivered via microvesicles. **(C)** CHO-native cells were incubated with free Stx2:Alexa555 and stained with DAPI. No fluorescence was seen in CHO-native cells that had been given Stx2:Alexa555 in free form. **(D)** DLD-1 cells were incubated with free Stx2:Alexa488. No fluorescence was seen in DLD-1 cells that had been given Stx2:Alexa488 in free form. Two independent experiments were performed and representative results are presented.

To investigate if Stx2, delivered within microvesicles, is taken up by Gb3-negative cells, fluorescently labeled microvesicles, containing Stx2:Alexa555, were isolated from stimulated platelets and incubated with CHO-native cells. Some of the labeled microvesicles were associated with the CHO-native cells within 4 h and a portion of the microvesicles were positive for Stx2:Alexa555 (Figure 4A). A similar experiment was performed with DLD-1 cells. Microvesicles containing Stx2:Alexa488 derived from HeLa cells were isolated and incubated with DLD-1 cells for 4 h. Z-stack images of the cells were acquired using three-dimensional structured illumination microscopy, revealing cytosolic presence of Stx2 (Figure 4B). As a control free Stx2:Alexa555 at the same concentration as in the microvesicles (as in Figure 4A) was used on CHO-native cells, showing a lack of cell association (Figure 4C). Similarly, free Stx2:Alexa488 could not be visualized within DLD-1 cells (Figure 4D). Quantification was performed as shown in Supplementary Figure 5.

### Uptake of Platelet-Derived Microvesicles by Gb3-Positive and Gb3–Negative CHO Cells

To determine if Gb3-positive and Gb3-negative cells take up equivalent amounts of microvesicles, CHO-Gb3 and CHO-control cells were incubated with blood cell-derived microvesicles, followed by analysis of CD41/CD61, platelet markers, in the microvesicle-containing supernatants. The median values of CD41/CD61 in the microvesicle suspensions after 2 h incubation were 84.1 ng/mL (range 69.9–100.1, *n* = 6) and 73 ng/mL (range 55.9–94.4, *n* = 6) for CHO-Gb3 and CHO-control, respectively, and after 24 h incubation 102.4 ng/mL (range 93.2–111.0, *n* = 3) and 97.2 ng/mL (range 93.2–112.2, *n* = 3), respectively, indicating that the cells took up equivalent amounts of microvesicles.

### Exogenous Gb3 Did Not Introduce Stx2 Toxicity in DLD-1 and CHO-Native Cells

**Figure 5:**
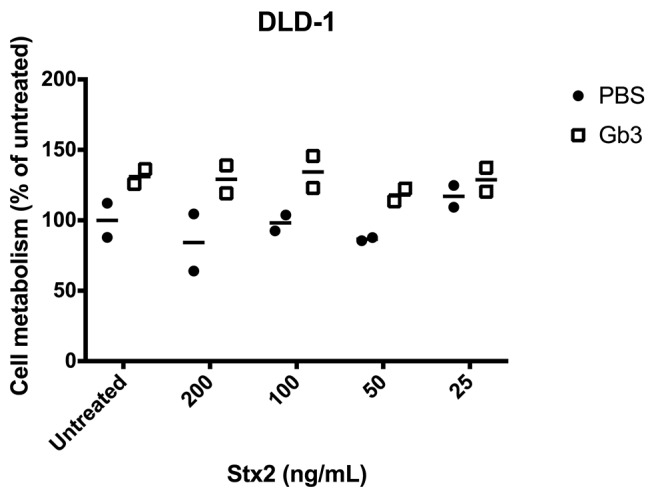
Exogenous administration of Gb3 did not introduce Stx2 susceptibility in DLD-1 cells. DLD-1 cells were incubated with Gb3-containing liposomes or PBS control followed by incubation with Shiga toxin 2 (Stx2) for 24 h. No significant difference in cell metabolism could be seen in PBS-treated or Gb3-liposome treated cells at any of the Stx2 concentrations. Median cell metabolism is denoted by the bar.

To test if exogenously administrated Gb3 could introduce Stx2-sensitivity, Gb3-negative DLD-1 or CHO-native cells were treated with exogenous Gb3-liposomes followed by incubation with Stx2 (range 25–200 ng/mL) for 24 or 48 h. Exogenous Gb3 did not introduce sensitivity to Stx2 in DLD-1 cells (Figure 5) or in CHO-native cells (data not shown). To confirm that the exogenously administrated Gb3 had indeed been incorporated into the cell membrane, Stx2 labeled with Alexa Fluor 488 was incubated with Gb3-liposome treated DLD-1 cells for 1 h. Fluorescent imaging showed that Stx2 was mainly located in the cell membrane (Supplementary Figure 7). Similar results were obtained when using CHO native cells showing Stx2 binding to the membrane (data not shown). As exogenous Gb3 did not introduce Stx2-sensitivity, experiments with Stx2-positive microvesicles were not performed.

## Discussion

Shiga toxin can be taken up by cells after binding to its receptor, Gb3, on the cell surface, or by cellular uptake of bacterial outer membrane vesicles (Bielaszewska et al., 2017) or host cell-derived microvesicles containing the toxin (Ståhl et al., 2015). If a cell lacks the toxin receptor it will not bind the toxin and will thus be resistant to the toxin's cytotoxic effect (Lindberg et al., 1987). We have, however, demonstrated that Gb3-negative cells could take up microvesicles containing the toxin (Ståhl et al., 2015). In this study we used three Gb3-negative cells, specifically CHO cells (native or CHO-control), PPMP (glycosylceramide synthase inhibitor)-treated HeLa cells as well as DLD-1 cells. We showed that CHO cells and DLD-1 cells could take up toxin-positive microvesicles but the cells did not respond with reduced viability (cell metabolism, CHO) or inhibited protein synthesis (CHO and DLD-1) to microvesicles containing Stx2 or to free Stx2. CHO cells transfected with A4GALT expressed the Gb3 receptor and became susceptible to toxin-positive microvesicles. Similarly, HeLa cells that were not treated with PPMP also responded to these microvesicles by significantly reduced cell metabolism and protein synthesis. Toxin contained in microvesicles utilized the retrograde transport pathway to inhibit protein synthesis, which could be reduced by the specific inhibitor Retro-2.1. We conclude that recipient cells that take up Stx2-positive microvesicles must possess endogenous Gb3 in order to become susceptible to the effects of the toxin and that Gb3 present in the microvesicles themselves was insufficient to promote cytotoxicity.

Studies from our group have shown that Stx2 circulates bound to blood cell-derived microvesicles and thereby reaches the kidney (Ståhl et al., 2015). Patients with EHEC-associated HUS were shown to have elevated levels of circulating microvesicles derived from platelets, neutrophils, monocytes, and red blood cells, and these vesicles were positive for Stx2. The toxin-positive microvesicles from blood cells were detected within kidney cells (Ståhl et al., 2015; Karpman et al., 2017). Stx2-positive blood cell-derived microvesicles exerted a toxic effect on glomerular endothelial cells. Only trace amounts of free toxin have been detected in the circulation of HUS patients (Brigotti et al., 2011). Thus, we assume that toxin delivery to the kidney within host cell-derived microvesicles may be one of the main mechanisms of toxin transfer from the intestine to the target organ (Karpman et al., 2017). It was therefore of interest to investigate if these toxin-containing microvesicles can be taken up by, and exert a cytotoxic effect, on Gb3-negative cells.

The microvesicles used herein were shed from blood cells that were stimulated with Stx2 and contained Stx2 within the vesicle. The toxin binds to Gb3 on the blood cells, is taken up and shed within microvesicles (Karpman et al., 2017). These microvesicles were shown here to be Gb3-positive and bind Stx2, as demonstrated by thin layer chromatography and Stx2 overlay. When incubated with Gb3-positive recipient cells the microvesicles were taken up and affected cell metabolism and viability by inhibiting protein synthesis. The microvesicles themselves must possess Gb3 in order to bind toxin but the role of the Gb3 receptor in the recipient cell in contributing to sorting of microvesicle content at early endosomes and retrograde transport of toxin is unclear. We could show that in Gb3-positive cells toxin delivered within microvesicles was transported by the retrograde route (an effect reduced by Retro-2.1). This is in line with previous publications showing that Stx2 delivered in microvesicles reaches the ER (Ståhl et al., 2015) and that Retro-2 blocks the retrograde transport of Stx (Stechmann et al., 2010). Presumably the glycolipid composition within lipid rafts present in intracellular membranes contributes to retrograde transport of Stx, and evidently lack of sufficient intracellular Gb3 abrogates this effect.

Cell viability was assessed using the redox Alamar blue assay and staining was indicative of the number of living cells. An interesting incidental observation was that cells exposed to microvesicles that did not contain Stx2 exhibited a higher cellular metabolism. CHO-Gb3 cells, incubated with microvesicles lacking Stx2, and CHO-control cells, incubated with microvesicles with and without Stx2, had a significantly higher cellular metabolism compared to untreated cells (Figure 1). A similar observation was noted regarding protein synthesis in which CHO-Gb3 cells treated with Stx2-negative microvesicles exhibited somewhat higher protein synthesis compared to untreated cells (Figure 2A). These experiments were performed in the absence of serum and we speculate that the increase in cellular metabolism and protein synthesis may depend on additional nutrients contributed by the microvesicle suspensions.

Stx that is bound to Gb3 not present in lipid rafts will be directed to lysosomes for destruction (Falguieres et al., 2001). We assume that some toxin-positive microvesicles taken up by cells may be routed to lysosomes for destruction, possibly more so in Gb3-negative cells. Reduced glucosylceramide was previously shown to affect lipid rafts in the ER and toxin translocation across ER membranes, thereby decreasing susceptibility to Stx (Smith et al., 2006). This could explain why Gb3-negative cells were resistant to the effects of toxin packaged within Gb3-positive microvesicles as the ER membranes in recipient cells should also possess a certain glycosphingolipid composition.

In this study we chose to work with the CHO cell line as these Gb3-negative cells have previously been shown to become sensitive to Stx when transiently transfected with the Gb3-synthase A4GALT (Keusch et al., 2000). To our knowledge this is the first time a cell line has been stably modified to become sensitive to Stx and together with the Stx-insensitive vector control they provide a good model for *in vitro* studies of the toxin. In these CHO-Gb3 cells the dominant glycosphingolipid appeared to be Gb4 (Supplementary Figure 1B). Stx2 may bind to both Gb3 and Gb4 (Gallegos et al., 2012). Even if cells express more Gb4, Stx2 may preferentially bind Gb3 as we could show in Supplementary Figure 2, and as has been described previously (Lingwood et al., 1987; Karve and Weiss, 2014). We cannot, however, rule out the possibility that both Gb3 and Gb4 are relevant for Stx2 sensitivity in the CHO-Gb3 cells. We used human HeLa cells because they have been used in numerous Stx-transport and toxicity studies and have been shown to become Stx-insensitive when treated with glucosylceramide inhibitors (Smith et al., 2006). DLD-1 cells are Gb3-negative human colorectal epithelial cells and were used because of their relevance for *in vivo* EHEC infections.

Gb3 is essential for a response to Stx in a murine *in vivo* model (Okuda et al., 2006). In Gb3-positive animals we observed that Gb3-negative cells could take up Stx2-containing microvesicles *in vivo* (Ståhl et al., 2015). Mice were inoculated with Stx2-producing EHEC and toxin-positive microvesicles were detected in the murine glomerular endothelium as well as within the glomerular basement membrane (Ståhl et al., 2015). In order for blood cell-derived microvesicles (with retained markers of the blood cell of origin) to reach the glomerular basement membrane they would need to pass through or between glomerular endothelial cells without emptying their content. The findings in the present study suggest that Stx2-positive microvesicles that are taken up by Gb3-negative cells do not affect cell viability. This could be due to lack of toxin release from vesicles and/or lack of retrograde transport. If some of the toxin is not released from vesicles in Gb3-negative cells this could promote passage through the cells to the basement membrane under conditions in which inflammatory mediators enhance cell and basement membrane permeability, such as during EHEC infection.

In summary, our findings suggest that uptake of Gb3- and Stx2-positive microvesicles is not sufficient to induce cell death and that the recipient cell must possess Gb3, and possibly a certain glycosphingolipid composition within its intracellular lipid microdomains, for Stx2 delivered within microvesicles to exert its toxic effect. Uptake of Stx2-positive microvesicles to Gb3-negative cells will not affect cell viability but may enable passage of the vesicles through the cells in an inflammatory microenvironment.

## Data Availability Statement

All data in this study are available from the corresponding author upon request.

## Ethics Statement

The studies involving human participants were reviewed and approved by Regional Ethics Review Board of Lund University. The participants provided their written informed consent to participate in this study.

## Author Contributions

KJ, AW, A-CK, AT, and AS designed and conducted experiments. DG contributed material. KJ, AW, A-CK, AT, AS, and DK analyzed data. KJ and DK wrote the manuscript. All authors read and approved the final manuscript.

## Conflict of Interest

The authors declare that the research was conducted in the absence of any commercial or financial relationships that could be construed as a potential conflict of interest.
