# Supplementary material for: Shiga Toxin-Bearing Microvesicles Exert a Cytotoxic Effect on Recipient Cells Only When the Cells Express the Toxin Receptor
Source: Front Cell Infect Microbiol. 2020 May 25;10:212. doi: 10.3389/fcimb.2020.00212 (PMC7261856; doi:10.3389/fcimb.2020.00212)
Supplement: Supplementary file 1 [file Data_Sheet_1.zip › Figure S1.pdf]

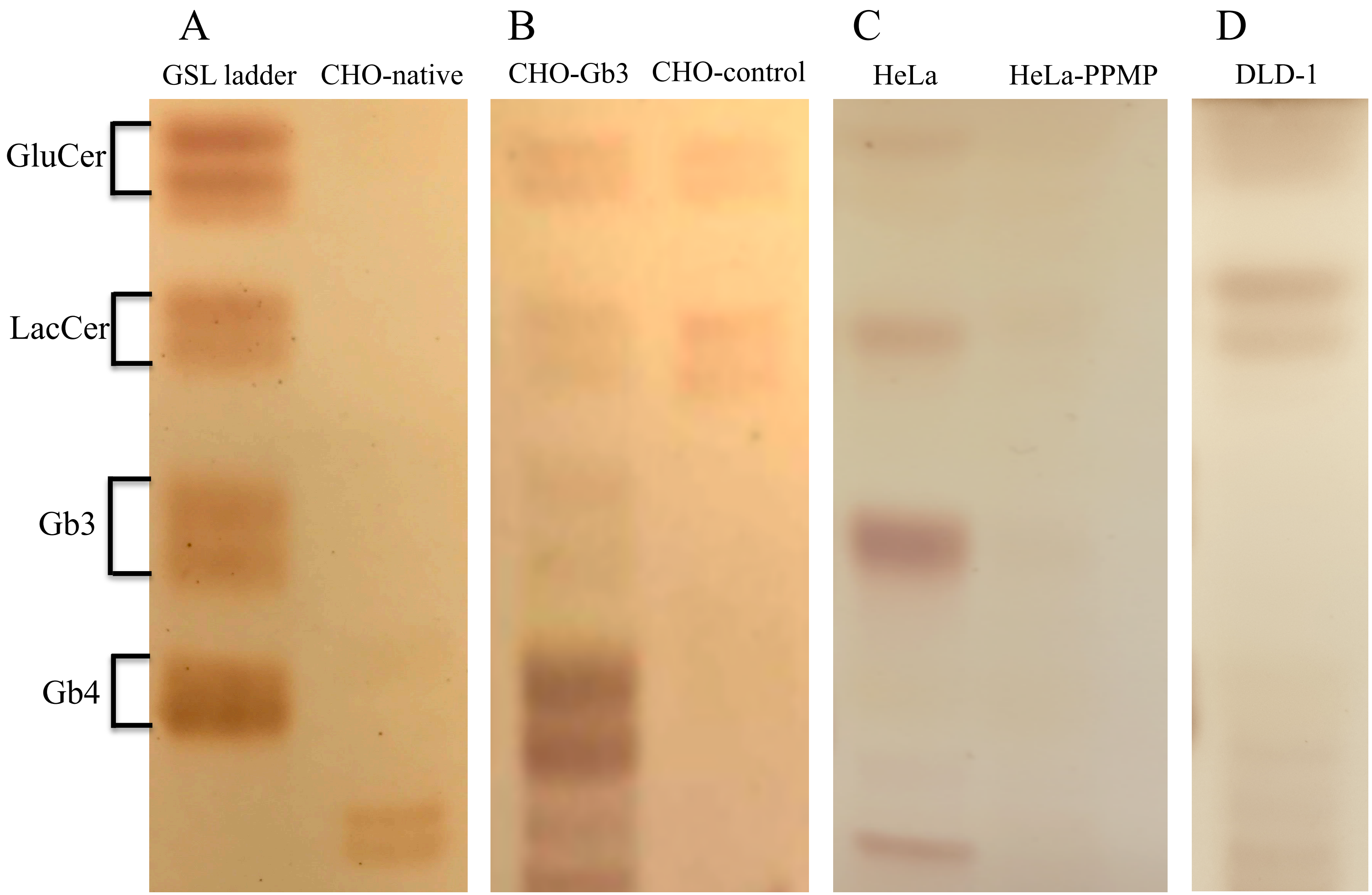


**Supplementary Figure S1: Orcinol staining of total glycosphingolipid extracts separated by thin layer chromatography.** Glycosphingolipids were identified using a predefined standard (GSL ladder) containing glucosylceramide (GluCer), lactosylceramide (LacCer), globotriaosylceramide (Gb3) and tetraosylceramide (Gb4). The glycosphingolipid content of (**A**) CHO-native, (**B**) CHO-Gb3 and CHO-control, (**C**) HeLa and HeLa-PPMP and (**D**) DLD-1 cells is presented.
