# Supplementary material for: Shiga Toxin-Bearing Microvesicles Exert a Cytotoxic Effect on Recipient Cells Only When the Cells Express the Toxin Receptor
Source: Front Cell Infect Microbiol. 2020 May 25;10:212. doi: 10.3389/fcimb.2020.00212 (PMC7261856; doi:10.3389/fcimb.2020.00212)
Supplement: Supplementary file 1 [file Data_Sheet_1.zip › Figure S2.pdf]

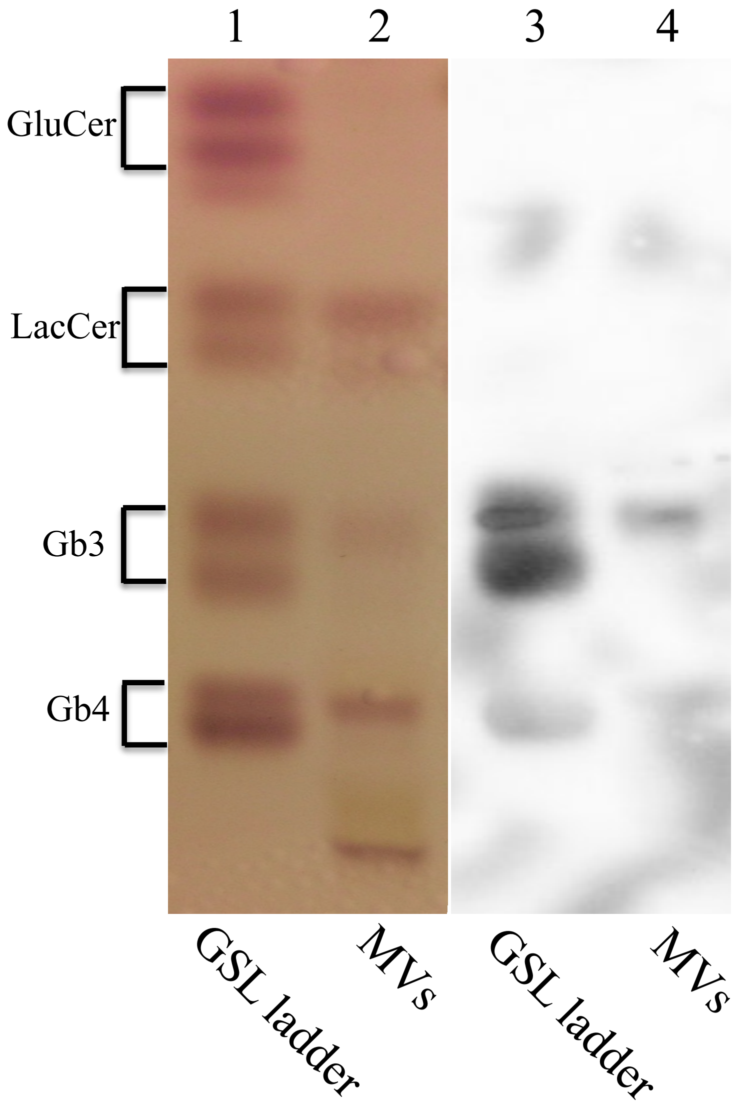

Supplementary Figure S2: Neutral glycosphingolipid content and Stx2-overlay of blood cell-derived microvesicles. The glycosphingolipid content of blood cell-derived microvesicles (MVs) was extracted and separated by thin layer chromatography together with a predefined glycosphingolipid standard (GSL ladder) containing glucosylceramide (GluCer), lactosylceramide (LacCer), globotriaosylceramide (Gb3) and tetraosylceramide (Gb4) (lane 1). The MV extract exhibited bands corresponding to LacCer, Gb3 and Gb4 (lane 2). Stx2-binding was visualized by Stx2-overlay (lanes 3 and 4) showing extensive binding to Gb3 and that the Gb3 in MVs binds Stx2 (lane 4).
