# Supplementary material for: Shiga Toxin-Bearing Microvesicles Exert a Cytotoxic Effect on Recipient Cells Only When the Cells Express the Toxin Receptor
Source: Front Cell Infect Microbiol. 2020 May 25;10:212. doi: 10.3389/fcimb.2020.00212 (PMC7261856; doi:10.3389/fcimb.2020.00212)
Supplement: Supplementary file 1 [file Data_Sheet_1.zip › Figure S3.pdf]

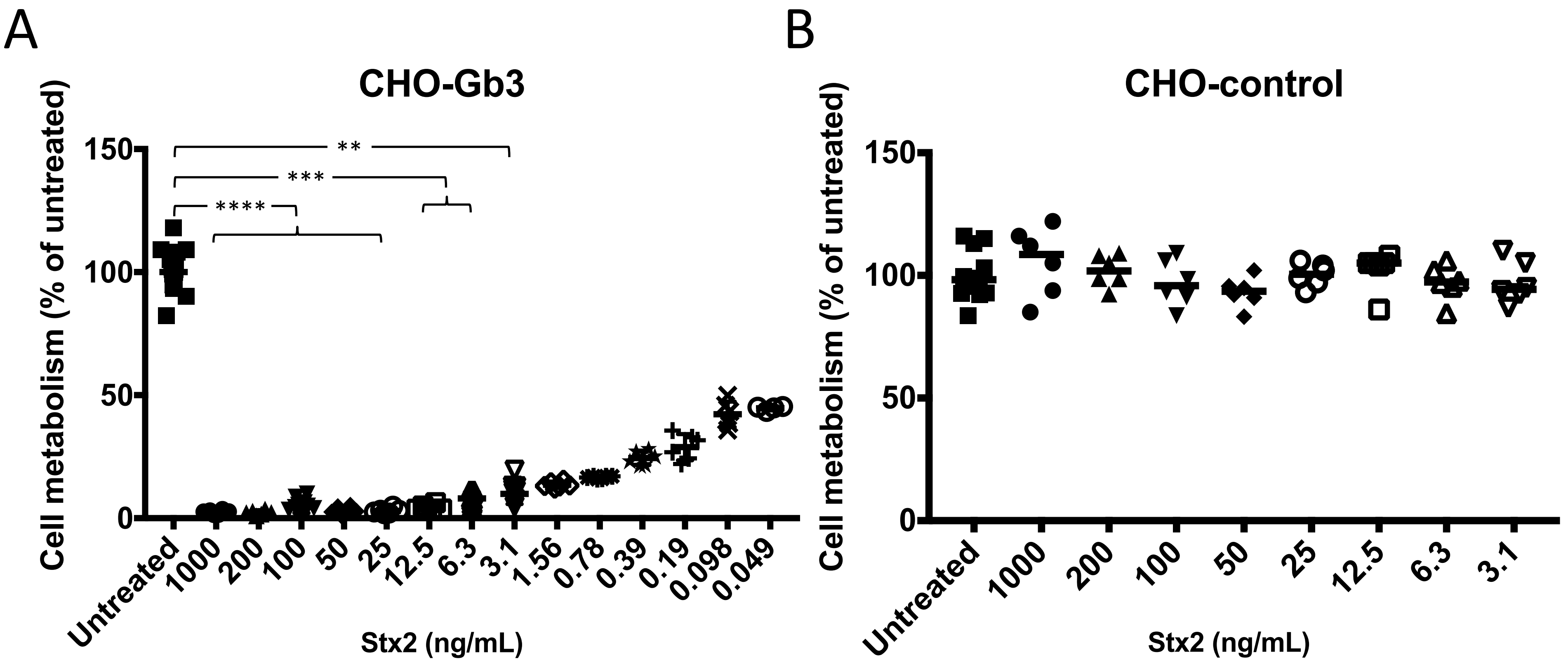

Supplementary Figure S3: CHO cells become sensitive to Stx2 upon A4GALT transfection

CHO-Gb3 and CHO-control cells were incubated with Shiga toxin (Stx2) and cell metabolism was measured. (**A**) A significant difference was seen in CHO-Gb3 cells between untreated and Stx2-treated cells in the range of 25 – 1000 ng/mL (P<0.0001), between untreated and Stx2-treated at 6.3 and 12.5 (P<0.001) and between untreated and Stx2-treated at 3.1 ng/mL (P<0.01) (2 independent experiments). (**B**) In CHO-control cells incubated with Stx2 no decrease in cell metabolism could be detected (one experiment). Median cell metabolism is denoted by the bar. **: P<0.01, ***: P<0.001, ****: P<0.0001, Kruskal-Wallis test.
