# Supplementary material for: Shiga Toxin-Bearing Microvesicles Exert a Cytotoxic Effect on Recipient Cells Only When the Cells Express the Toxin Receptor
Source: Front Cell Infect Microbiol. 2020 May 25;10:212. doi: 10.3389/fcimb.2020.00212 (PMC7261856; doi:10.3389/fcimb.2020.00212)
Supplement: Supplementary file 1 [file Data_Sheet_1.zip › Figure S4.pdf]

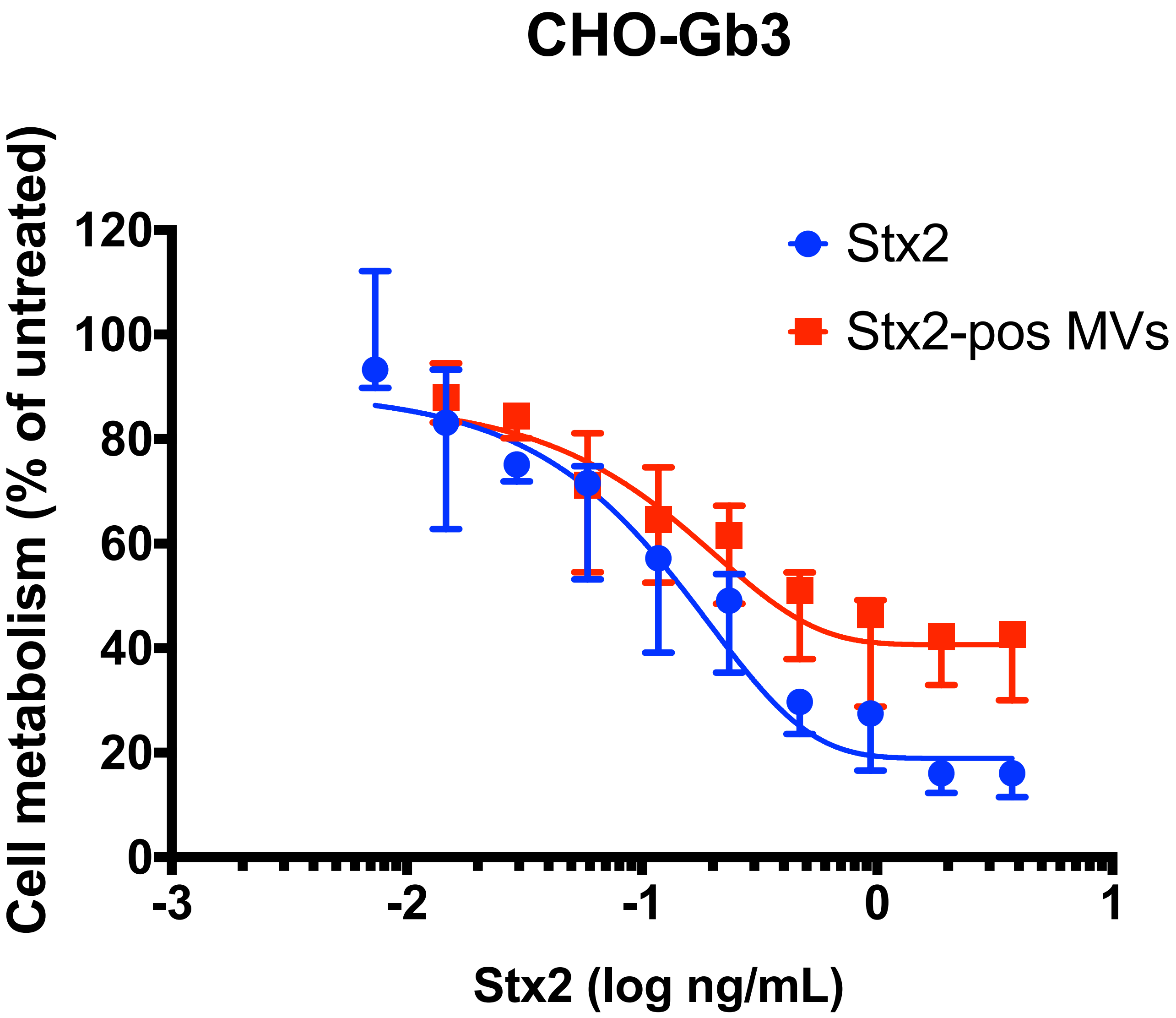

Supplementary Figure S4: Dose-response curves of Stx2-positive microvesicles and free Stx2. CHO-Gb3 cells were treated with increasing concentrations of Stx2-positive microvesicles (Stx2-pos MVs, n=5) and free Stx2 (n=3). The IC_{50} value of Stx2-pos MVs was 0.088 ng/mL and the IC_{50} value of free Stx2 was 0.11 ng/mL. Median and range are denoted. Two independent experiments are presented.
