# Supplementary material for: Shiga Toxin-Bearing Microvesicles Exert a Cytotoxic Effect on Recipient Cells Only When the Cells Express the Toxin Receptor
Source: Front Cell Infect Microbiol. 2020 May 25;10:212. doi: 10.3389/fcimb.2020.00212 (PMC7261856; doi:10.3389/fcimb.2020.00212)
Supplement: Supplementary file 1 [file Data_Sheet_1.zip › Figure S5.pdf]

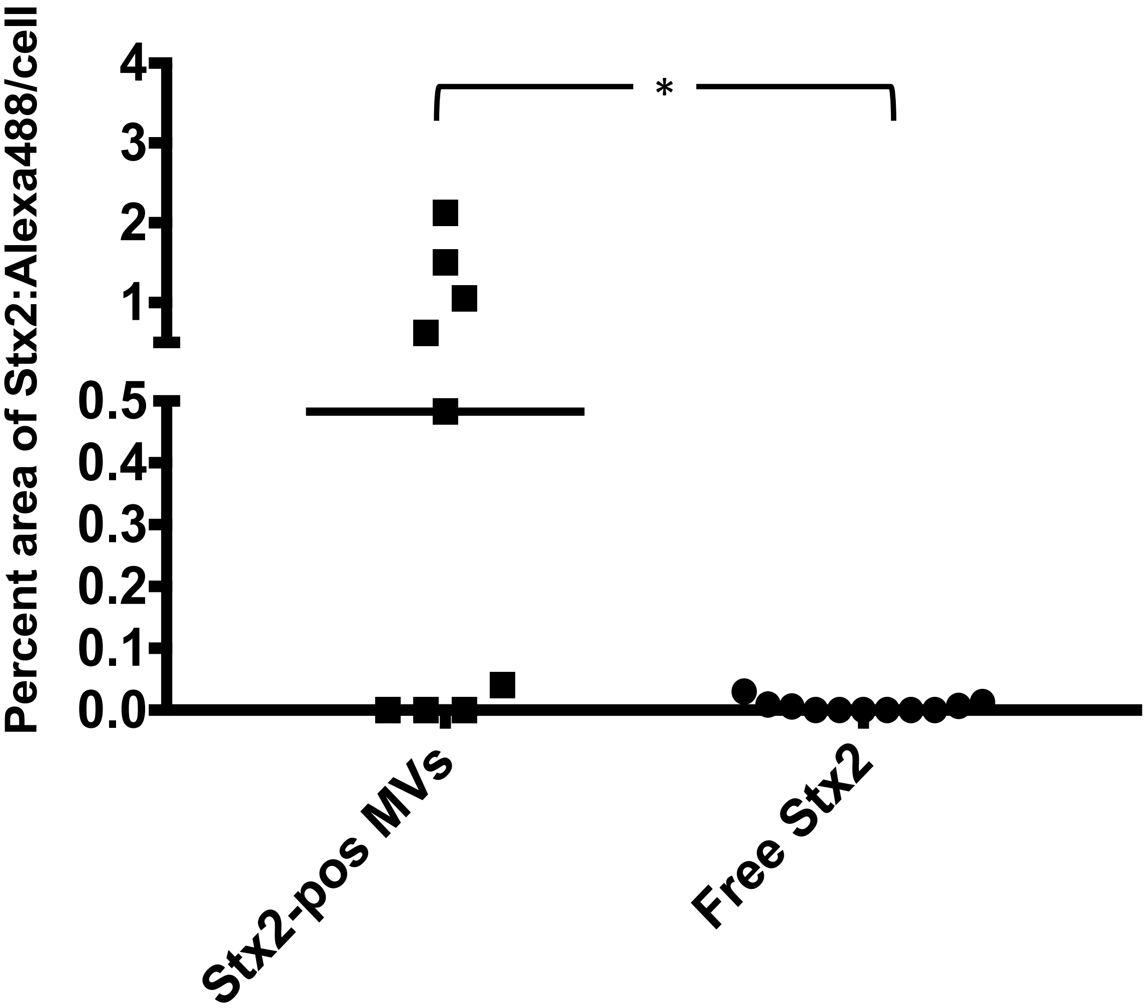

Supplementary Figure S5: Quantification of Stx2:Alexa488 uptake in DLD-1 cells

DLD-1 cells were incubated with HeLa cell derived Stx2:Alexa488 positive microvesicles (Stx2-pos MVs) or with the corresponding amount of free Stx2:Alexa488, imaged using fluorescence microscopy and the green fluorescence in each cell was quantified. A significantly larger area was positive for Stx2:Alexa488 in the DLD-1 cells that had been incubated with Stx2-pos MVs compared to free toxin. The median is denoted by the bar. *: $P<0.05$, Two-tailed Mann Whitney U test.
