# Supplementary material for: Shiga Toxin-Bearing Microvesicles Exert a Cytotoxic Effect on Recipient Cells Only When the Cells Express the Toxin Receptor
Source: Front Cell Infect Microbiol. 2020 May 25;10:212. doi: 10.3389/fcimb.2020.00212 (PMC7261856; doi:10.3389/fcimb.2020.00212)
Supplement: Supplementary file 1 [file Data_Sheet_1.zip › Figure S6.pdf]

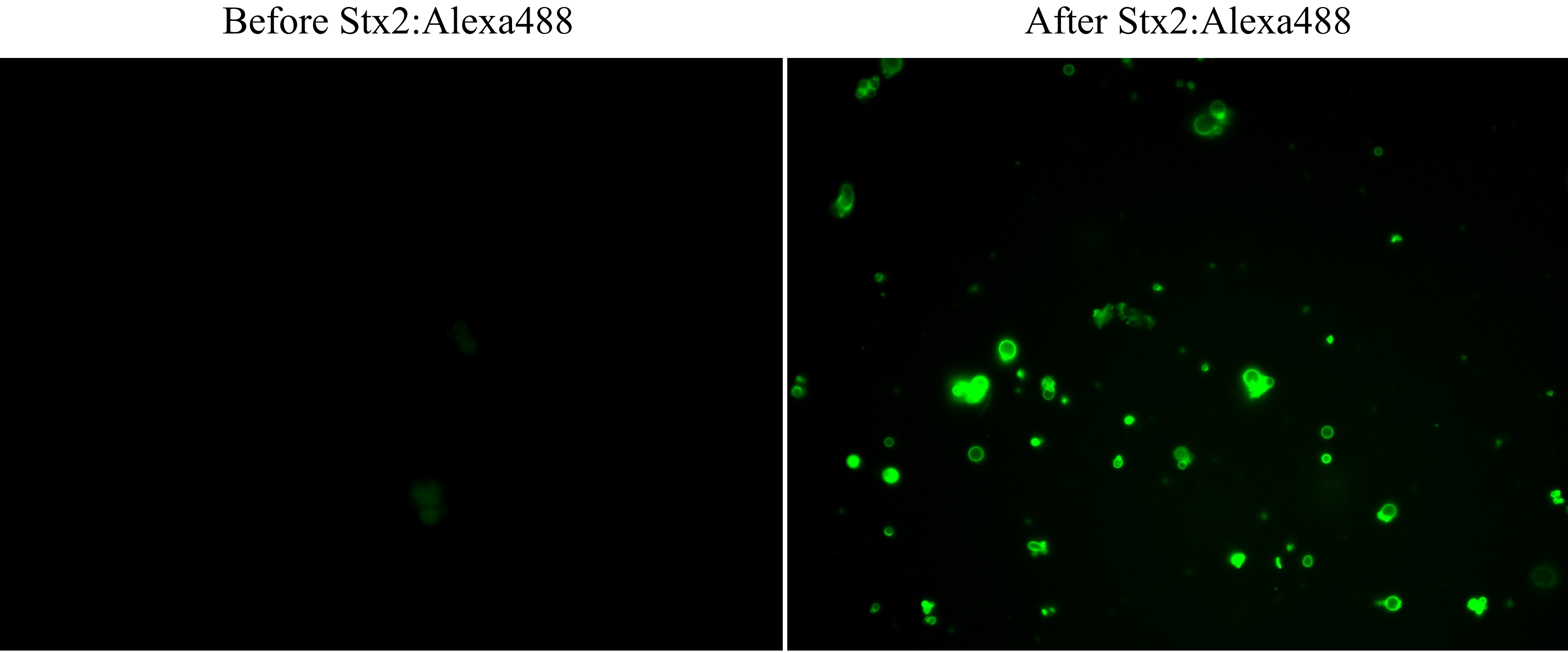

Supplementary Figure S6: Stx2 binding of Gb3-liposome. Liposomes consisting of globotriaosylceramide, phosphatidylserine and phosphatidylethanolamine in equal portions before (left) and after (right) administration of Stx2:Alexa488. Visualized by an Axio Observer.A1 fluorescence microscope.
