# Supplementary material for: Shiga Toxin-Bearing Microvesicles Exert a Cytotoxic Effect on Recipient Cells Only When the Cells Express the Toxin Receptor
Source: Front Cell Infect Microbiol. 2020 May 25;10:212. doi: 10.3389/fcimb.2020.00212 (PMC7261856; doi:10.3389/fcimb.2020.00212)
Supplement: Supplementary file 1 [file Data_Sheet_1.zip › Figure S7.pdf]

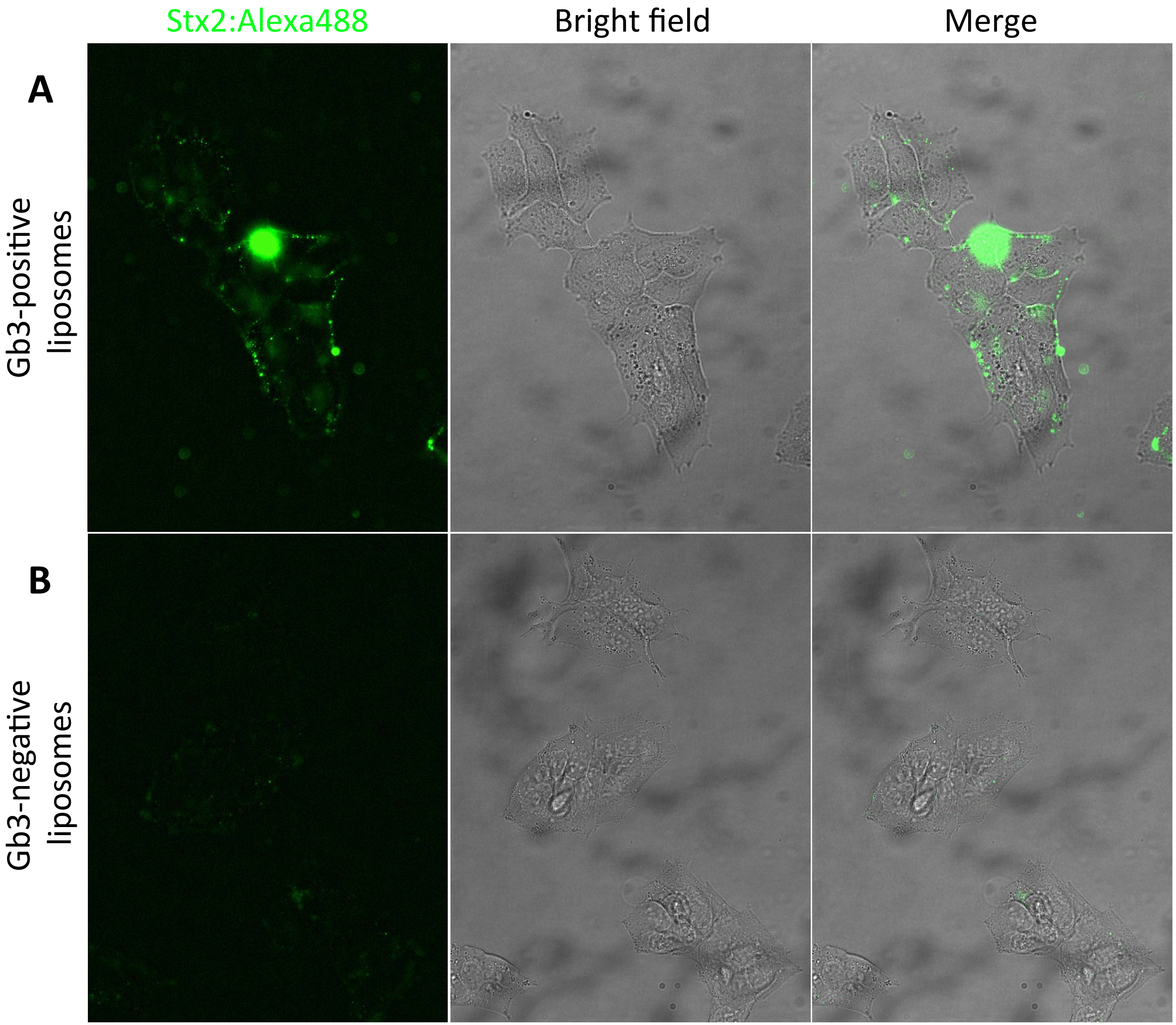

Supplementary Figure S7: Stx2 binding to DLD-1 cells treated with Gb3-positive and Gb3-negative liposomes. (A) Stx2:Alexa488 was administrated to DLD-1 cells that had been incubated with Gb3-positive liposomes. The bright field image shows 13 cells. Toxin binding was seen mainly along the membrane of the cells, as visualized in the merged panel to the right. (B) Stx2:Alexa488 was administered to DLD-1 cells that had been incubated with Gb3-negative liposomes. The bright field image shows 11 cells. No Stx2 binding was observed. Images were taken with an Axio Observer.A1 fluorescence microscope. The image is representative of visualization of 4 fields.
